# Makerere's contribution to the development of a high impact HIV research population-based cohort in the Rakai Region, Uganda

**DOI:** 10.4314/ahs.v22i2.7S

**Published:** 2022-08

**Authors:** Edward Nelson Kankaka, Fred Nalugoda, David Serwadda, Fredrick Makumbi, Maria J Wawer, Ronald H Gray, Thomas C Quinn, Steven J Reynolds, Gertrude Nakigozi, Tom Lutalo, Godfrey Kigozi, Nelson K Sewankambo, Joseph Kagaayi

**Affiliations:** 1 Rakai Health Sciences Program; 2 Makerere University College of Health Sciences, School of Public Health; 3 Makerere University College of Health Sciences, School of Medicine; 4 Johns Hopkins Bloomberg School of Public Health; 5 Johns Hopkins University School of Medicine; 6 Division of Intramural Research, National Institute of Allergy and Infectious Diseases, NIH; 7 Uganda Virus Research Institute

**Keywords:** Makerere, high impact HIV research, Rakai Region, Uganda

## Abstract

Longitudinal population-based cohort studies can provide critical insights on temporal, spatial and sociodemographic changes in health status and health determinants that are not obtained by other study designs. However, establishing and maintaining such a cohort study can be challenging and expensive. Here, we describe the role of Makerere University in the development and conduct of such a cohort. We chronicle the first academia-led reports of HIV in East Africa; how this led to initiation of the Rakai Community Cohort Study in 1988, the first and oldest HIV cohort in sub-Saharan Africa; its impact on HIV prevention, care and treatment; how the cohort has been maintained; and opportunities, challenges, and future directions including non-communicable diseases.

## Discovery of the first cases of HIV in Rakai, Uganda

A war between Uganda and Tanzania ended in 1979 with the overthrow of the Idi Amin government; this led to disputed elections in 1980 and the eventual outbreak of the Uganda civil war of 1980–1986. By 1982, reports began emerging of cases of an unknown disease in Rakai, a Ugandan district bordering Northern Tanzania and neighboring Masaka district. The information was passed on to the Uganda Ministry of Health which dispatched a team of senior doctors to investigate. They reported that these cases were likely to be usual diseases such as tuberculosis presenting with gastrointestinal manifestations. Nonetheless, Dr. Anthony Lwegaba, a young Medical Officer at the Kalisizo Health Center, Rakai, reached out to colleagues at Mulago Hospital, Makerere Medical School. Subsequently, a medical research team travelled to Rakai and Masaka districts. The team was comprised of Nelson K. Sewankambo, David M. Serwadda and Roy D. Mugerwa, all junior doctors at Mulago at the time; and J.W Caswell and Ann C. Bailey, senior British doctors working in Mulago Hospital and the University of Zambia in Lusaka, respectively (Ann C. Bailey had been working on Kaposi sarcoma). Nelson, David, and Roy wanted to better understand the disease. They secured donations of a few syringes, gloves and test tubes from Mulago Hospital, the Uganda Cancer Institute, and the Uganda Virus Research Institute (the civil war was still in progress, and basic supplies were difficult to come by), and Dr. Caswell asked the British embassy to lend them a Land Rover. Thus, without any formal research support, they headed south-west to Masaka Hospital and rural Rakai District.

The patients in Rakai and Masaka had intermittent fevers and moderate to severe diarrhea, an irritating skin rash, and were weak and extremely wasted. Local attendants were at a loss – they were having to resort to using mpombo (steamed soft banana leaves) as nappies for the patients with diarrhea. There were many fresh graves around households, and multiple afternoon burial processions were a common sight every day. The local people called the condition “slim disease” because of the extreme weight loss that patients manifested. They suggested that cross-border movements by military personnel during the earlier Uganda-Tanzania war, as well as traders involved in illicit trade brought this disease from Tanzania to Uganda. Uganda was undergoing severe economic hardship because of the repeated wars and smuggling from Tanzania was rampant across Lake Victoria and along dilapidated roads. Because many of the early patients were traders who frequently crossed the borders, Rakai residents theorized that the former engaged in commercial sex in Tanzania and were bewitched or cursed by medicine men in that country. In addition, some people thought the disease was a punishment from God for the illicit trade.

The research team identified selected patients with these symptoms at Kalisizo Health Center and Masaka Hospital and visited the homes of some of these individuals. The team took medical histories, examined in-hospital medical and surgical patients, and collected biological samples that included sputum, urine, stool, and blood. They noted similarities between these cases and those reported among homosexual men in North America at the time. The paradox was that the Uganda patients were heterosexual men and women. The cases in North America were called the Acquired Immune Deficiency Syndrome or AIDS, whose cause had been identified as the Human T-cell Leukemia Virus type 3 or the Lymphadenopathy-Associated Virus (HTLV-III/LAV)^1^,^2^, later renamed Human Immune deficiency Virus type-1 or HIV-1 3. Through Dr. Caswell, Dr. Robert Downing at the Uganda Ministry of Health / Uganda Virus Research Institute agreed to send these samples to the Public Health Laboratory in Porton Down, Salisbury, UK for testing where 63/71 samples tested positive for HTLVIII/LAV. This constituted the first report of HIV in East Africa, published in The Lancet^4^.

The Uganda civil war ended about three months later in January 1986, but the new government had a double challenge – rebuilding the country's dilapidated infrastructure and economy; and grappling with an untreatable and fatal new disease.

## Establishment of the first and oldest population-based HIV cohort in Africa, since 1988

Nelson and David wrote proposals to several funding agencies including the WHO and the US Centers for Disease Control (CDC) and received encouragement but no funding. Eventually after two years, Columbia University learnt of the Rakai reports and of the frantic search for funding, and asked Maria J. Wawer, a physician/epidemiologist to travel to Uganda to check out the potential for a small HIV/AIDS project, using a modest USAID grant left unexpended from a reproductive health project. Initially skeptical regarding research possibilities in Rakai, Maria was eventually convinced by the determination of David and Nelson, and a trip to Rakai.

*“So, we wrote a proposal for the use of the remaining USAID funds at Columbia University [$200,000 US dollars], got the go ahead, and the great adventure began*,” says Maria.

The “Rakai Project” started in 1988 and was boosted by funding from the NIH in 1989 to study the dynamics of HIV spread and its impact. The project was later re-named the Rakai Health Sciences Program (RHSP) in 2004 and continues as such to the present day. The first Rakai cohort included 21 randomly selected community clusters of about 30 households each along main roads, secondary roads and rural areas of Rakai (current day Rakai, Kyotera, and Lyantonde districts); and a total of around 1292 individuals who responded to interviews and provided blood samples.

*“There were no telephones or banks in Rakai. Money would be carried in sacks and quickly loaded into the land rover behind the bank building in Kampala to avoid curious eyes. In Rakai, we would pend the night at the Milano South View Inn in Kyotera Town, a very interesting place, and would pin blood samples with a hand-centrifuge under a single 40-watt light bulb dangling from the ceiling*,” recalls Nelson.

**Figure 1:**
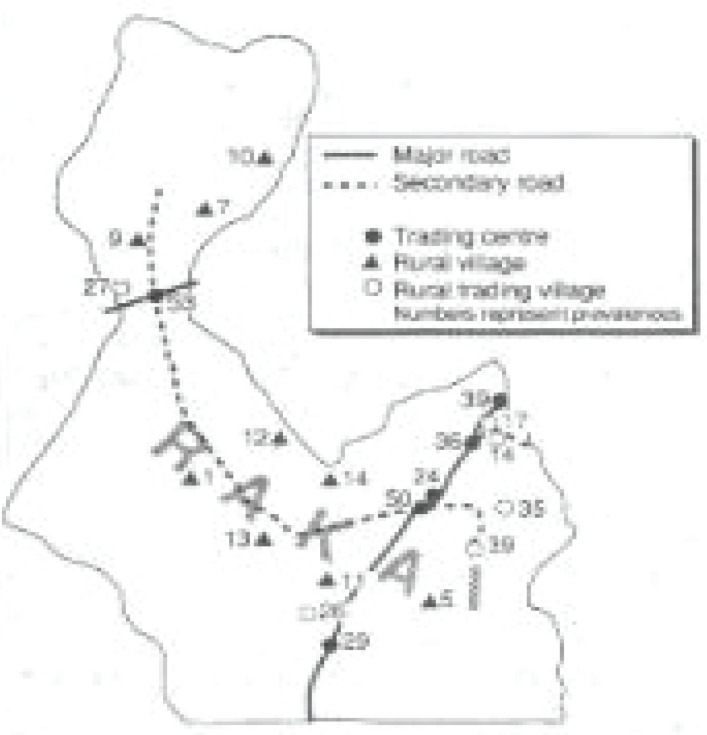
The first descriptions of HIV prevalence in the Rakai cohort (Maria Wawer et al, BMJ, 1991)

This work resulted in the first quantification of an HIV prevalence of up to 52.8% in some communities, as well as descriptions of the pattern of community spread - from main road trading centers, through intermediate trading villages, to rural agricultural villages (Figure 1)^5^. These data indicated that there was a public health emergency in the region.

**Figure 2:**
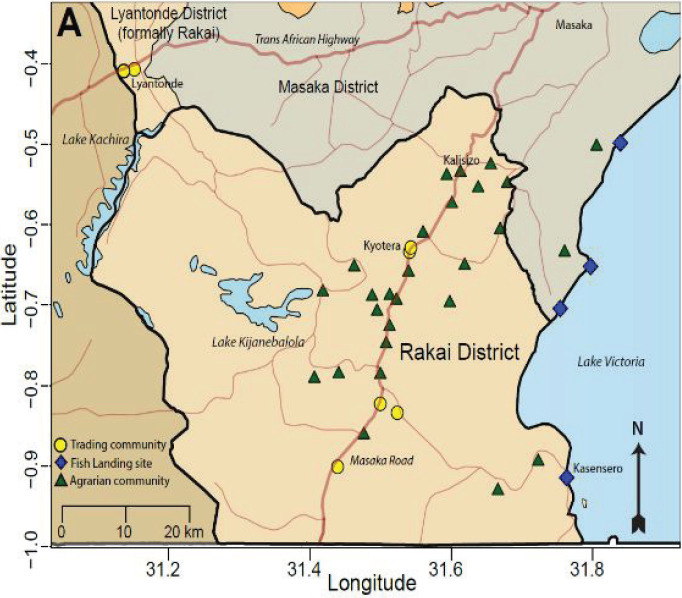
The modern Rakai Community Cohort Study

In the following years, additional funding from multiple funding agencies and foundations, including NIH, CDC, the World Bank, the Ugandan Ministry of Health, the Rockefeller Foundation, the Gates Foundation, the Walter Reed Army Institute of Research/Henry Jackson Foundation, and the Doris Duke Charitable Foundation enabled expansion of the Rakai project with establishment of a lab, inclusion of new communit clusters, provision of counseling services, and new HIV/STD studies nested within the cohort. The cohort in its current form (Figure 2) was reorganized and further expanded in 1994 and has since included comprehensive sociodemographic, behavioral and health interviews, and collection of biospecimens for assessment of HIV, other sexually transmitted infections (STIs), opportunistic infections, and more recently, non-communicable diseases once every 18 months. At the close of 2020, the Rakai Community Cohort Study (RCCS), included over 22,000 individuals aged 15 to 49 years old from 40 agrarian, trading, or fishing communities. The upper age limit was lifted in 2021 to also expand surveillance of emerging non-communicable diseases.

## Impact of the Rakai cohort on HIV prevention, treatment, and care policy

**Figure 3:**
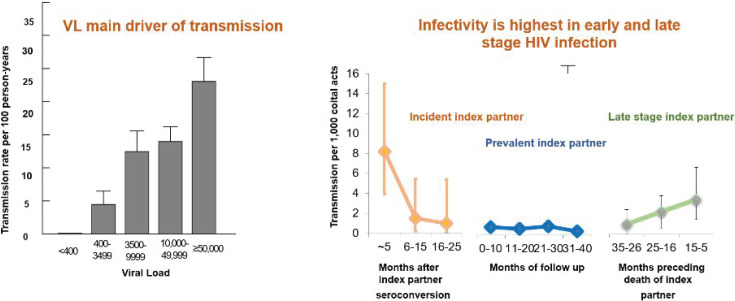
HIV Viral Load, Stage of Infection and Transmission: Analyses among discordant couples (Quinn et al. N Engl. J Med 2000, Wawer et al, JID, 2005)

The cohort has thrived over the years and has had a profound impact on our understanding of HIV epidemiology, spatial distribution, modes of prevention, treatment, and care globally. Since the early 1990s, cohort data provided numerous insights into the effects of HIV on population-level demography, modes of transmission and risk factors^6^–^11^. Between 1994–1998, the RHSP nested a community-randomized trial to test the effect of mass treatment of STIs on HIV incidence. Although the intervention significantly reduced the prevalence of STIs, it did not reduce HIV incidence^12^. The latter negative finding of the trial was disappointing (though this finding was later replicated in multiple other international studies), the data and biospecimen repository helped generate important hypotheses with high-impact research findings. Early viral load quantification in this cohort pointed to viremia level as a main driver of HIV transmission and that viral load was highest in the early and late stages of HIV infection (Figure 3)^13^,^14^. In addition, a nested study on maternal and infant health observed multiple benefits for both the women and their infants from the low cost, feasible mass STI treatment used in the trial^15^.

Zidovudine (AZT), the first proven treatment for HIV to markedly suppress HIV viraemia was available to patients in the US and other high-income countries as of 1987 16,17. However, AZT and other drugs developed subsequently, did not become available in Uganda until 2002, and initially only at the Joint Clinical Research Center in Kampala (using generic AZT imported from India). The drug only became available in Rakai in 2004, once The President's Emergency Plan for AIDS Relief (PEPFAR) began to provide HIV drugs to selected countries around the world. Notably, RHSP was extensively involved in the Drug Access Initiative to make HIV treatment available in sub-Saharan Africa. The work on viral load and heterosexual transmission of HIV-1 by stage of infection in the Rakai cohort (Figure 3)^13^–^15^, led to the concepts of ‘treatment as prevention’, ‘universal test and treat’, and ‘undetectable=untransmissible’ which were confirmed by trials such as HPTN 052^18^. These concepts are now standard practice, including routine viral load monitoring of patients initiated on treatment. The RHSP has also evaluated more affordable assays for viral load monitoring in resource-limited settings^19^.

In 2004–2007, an NIH-funded clinical trial proved the effectiveness of Voluntary Medical Male Circumcision (VMMC)^20^,^21^ along with two other clinical trials in Kenya and South Africa 22,23, which confirmed that the procedure was safe, acceptable and reduced the risk of male HIV acquisition by over 50%. VMMC also reduced the risk of male acquisition of other STIs 24,25, including in HIV-positive men^26^. The WHO and the Joint United Nations Program on HIV/AIDS (UNAIDS) recommended VMMC for HIV prevention in countries with high HIV burdens and low male circumcision prevalence 27. It is now an integral part of HIV programs in over 14 countries in sub-Saharan Africa. Since the Rakai cohort was also the only of the threerials to include female partners, it was also able to show that VMMC was highly acceptable to men and their partners^28^,^29^, and significantly reduced male transmission of multiple STIs to these partners^30^.

Through the cohort, more attention was drawn to the need for enhanced treatment of pregnant women and prevention of mother to child transmission (PMTCT)^7^. Effective modalities of delivering antiretroviral therapy to pregnant mothers and their infants were suggested^31^ and the benefit of continued breast feeding of HIV-exposed babies was demonstrated^32^, as well as the potential for integration of contraception services in HIV care for the mothers^33^.

The Rakai cohort has also provided a platform to evaluate the impact of HIV combination interventions, including VMMC and antiretroviral therapy. In 2017, the RHSP documented the first population-level impact of combination HIV prevention including a 42% reduction in HIV incidence within about 10 years^34^ in the general population and 48% decline (within 5 years) in hyper-endemic fishing communities^35^. The RHSP, based on cohort has conducted multiple implementation research studies to determine how to improve the availability and acceptance of combined HIV prevention^36^,^37^. Since 2016, RHSP has supported a comprehensive HIV service program (beyond VMMC and ART) across 12 districts of the Masaka region (including Rakai) with funding from PEPFAR through CDC-Uganda. The program includes prevention services such as Pre- and Post-Exposure prophylaxis (PrEP and PEP), DREAMS—an HIV prevention program among adolescent girls and young women, programs to reach key and priority populations with treatment, and program to mitigate effects of intimate partner violence and to protect children.

An ongoing collaboration with the PANGEA-HIV consortium, funded by the Bill and Melinda Gates Foundation, which includes multiple southern and eastern African cohorts, is assembling the most comprehensive data and analyses on HIV phylogenetics on the continent. With the Rakai cohort as the largest biospecimen contributor, the study has shown that “hot spots” such as fishing communities do not spread HIV to the general populations and that “super spreaders” do not play a major role in transmission^38^. Recent epidemiologic and phylogenetic data from the Rakai cohort and other cohorts in sub-Saharan Africa show that in a generalized African epidemic “many infect few”.

By 2022, nearly 600 publications have come from this cohort and have been cited more than 20,000 times. The cohort is expanding to follow-up Rakai cohort participants who migrate to major urban areas in Uganda (Kampala and Masaka) to determine how such migration affects access to and use of HIV prevention and treatment, and importantly, any changes in NCD status (e.g., hypertension, asthma, and related conditions).

*“We owe this small community of committed researchers a debt of gratitude for all the knowledge they have given us, from their post on the front lines of this terrible scourge. Paraphrasing Sir Winston Churchill, never was so much owed by so many to so few!”* Roger I Glass, Director, Fogarty International Center^39^.

## Partnerships to support the cohort and science

The role of partnerships between the RHSP and its population-based cohort, Makerere University, the Ugandan MOH, and multiple international entities is evident from David and Nelson's early efforts to mobilize multidisciplinary, national, and international teams to address the HIV problem in Uganda. The strategic partnerships built over the years have been invaluable in accelerating progress and maximizing local and international impact. RHSP now has co-investigators from multiple institutions including Makerere University, Uganda; Johns Hopkins University, USA; Center for Disease Control-Uganda; Columbia University, USA; Karolinska Institute, Sweden; University of Western Ontario, Canada; University of Toronto, Canada; and the Oxford University Big Data Institute, UK. It has been designated an International Center for Excellence in Research by the National Institute of Allergy and Infectious Diseases, NIH, USA, which greatly facilitates research collaboration with NIH Intramural Scientists. It is also an Implementing Partner for the PEPFAR program through CDC in Uganda. Multiple Ugandan scientists trained and mentored through the RHSP have in turn contributed to research, research training, public health education and mentoring laboratory sciences training and scholarship at Makerere and other Ugandan and foreign universities – including professors. Very importantly, the RHSP and the cohort provide a rich opportunity for researchers to gain higher degrees from Ugandan and international universities, using cohort data, and returning to a vibrant research environment in Uganda. With Gates Foundation and NIH support, among others, a research field station complex was built with a state-of-the-art clinic, laboratory, data management center, secure internet support, information technology equipment, biorepository, power backup infrastructure, research offices, transport fleet equipment and management, human resource and grant and finance management, conference and training facilities, and limited residential facilities for visiting researchers and some trainees.

## Opportunities, challenges, and the future

From small beginnings, the Rakai cohort in rural South-Central Uganda has been invaluable in fostering discoveries and providing information critical for HIV control that we had not envisioned in the early years of the epidemic. When the Makerere University researchers and colleagues from its partner institutions travelled to Rakai and Masaka districts in the early 1980's to research on “Slim” disease, they never imagined that they were setting a foundation for a future vibrant research institution in rural Africa with potential for global impact and that it would be in existence for nearly 35 years later. Given the physical and human infrastructure that exists now, the future is extremely bright and there are opportunities for expansion and greater impact in the region and globally through research, service delivery, education, and research capacity building. In the absence of an HIV vaccine and a cure - which the RHSP is increasingly researching 40–43, we are yet to close the chapter on HIV We must remain vigilant lest the gains made be lost. The epidemic must be consistently measured in the population and its ever-changing determinants characterized. The Rakai Cohort data have been made publicly available to other researchers on a dashboard^44^. Also, as people live longer and lifestyles and the environment change, non-communicable diseases (NCDs) are starting to emerge in the population and the cohort provides the infrastructure to study the epidemiology, test interventions and evaluate the population-level impact of these interventions on NCDs.

Longitudinal cohorts like the Rakai cohort require time and long-term commitment and are difficult to sustain financially. The lack of core cohort-specific funding which would ensure a stable research human resource is an enduring problem. Equally challenging is the long-term sustenance of the research infrastructure that has been developed over the years, largely dependent on winning foreign grants.

*“But the future is promising. It is now more than 30 years and the cohort is still alive and well. I am optimistic it will be sustained longer*,” says Nelson.

Going forward, the RHSP needs to establish an even stronger partnership with Makerere (and other Ugandan universities) with well-defined roles and responsibilities of each party. Ugandan institutions especially Makerere University can tap the research opportunities from the accumulated data, mentoring and potential partnerships. Local grants writing capacity needs to increase. There should be more research output from Ugandan and African students and researchers. RHSP should aim to become a stronger educational and research-training hub – hosting more doctoral, post-doctoral and visiting students and scientists with prolonged stay to increase research output and visibility. Makerere and other universities in Uganda and Africa should make efforts to acquire financial resources to foster and support inquisitive young minds – the story of RHSP started with such inquisitive young minds. Finally, the biggest testimony of Rakai will always remain the improved lives of the local population who participate in the research and are the primary recipients of Rakai's services.

*“We were asked – why go to Rakai [a miserable place then], are you born there? But we came. We volunteered our time. We did the first research without a budget. We started small, but were determined*,” concludes Nelson.
